# Selective Caries Removal: A Case Report with 21-Year Follow-Up

**DOI:** 10.1155/2024/3166087

**Published:** 2024-07-14

**Authors:** José Carlos Pettorossi Imparato, Kelly Maria Silva Moreira, Suzana Cavalcanti Monteiro de Oliveira, Sandra Regina Echeverria Pinho da Silva, Daniela Prócida Raggio

**Affiliations:** ^1^ Graduate Program in Pediatric Dentistry Institute and Research Center São Leopoldo Mandic Sao Leopoldo Mandic Faculty, Campinas, SP, Brazil; ^2^ Department of Pediatric Dentistry Pontifical Catholic University of Campinas, Campinas, SP, Brazil; ^3^ Orthodontics and Pediatric Dentistry Department University of São Paulo, São Paulo, SP, Brazil

## Abstract

Dental caries remains the most prevalent chronic disease globally, and its management should adhere to the principles of minimal intervention dentistry. This study reports a series of successful cases involving the selective removal of carious tissue in permanent molars, with follow-up periods of up to 21 years. Six permanent molars with severe and deep carious lesions were treated with selective caries removal and restored with high-viscosity glass ionomer cement. Clinical examination revealed that the teeth were free from pain and sensitivity. Follow-up assessments were conducted at 7 and 21 years posttreatment. Treatment success was defined by the absence of clinical and radiographic signs, symptoms of pulp alterations, and lesion arrest. Successful minimally invasive approaches were observed with selective removal of carious tissue and maintenance of pulp vitality for up to 21 years.

## 1. Introduction

Dental caries is the most prevalent noncommunicable disease worldwide, generating substantial direct and indirect costs, and is considered a public health problem [1]. Restorative treatment of deep carious lesions in young permanent molars presents a challenge for dentists, as complete removal of caries can expose the pulp tissue, necessitating more complex treatments, such as an endodontic treatment [2], or even early tooth loss [3].

The caries treatment options have significantly changed over time [2]. Traditionally, the management of deep carious lesions involves complete removal of carious tissue (CRCT); however, with the advent of minimally intervention dentistry, selective removal of carious tissue (SRCT) has been advocated [4]. SRCT allows dentin repair, prevents tissue loss, and facilitates conservative treatment of deep carious lesions [5]. Contrary to stepwise excavation (S.E.), which removes the carious tissue in two stages, a single clinical session is a current trend in treating carious lesions [6], because after sealing the cavity, tissue remineralization, bacterial reduction, and histological reorganization of the dentin occur [7].

Systematic reviews of the literature have concluded that CRCT can be considered an invasive technique with a higher risk of pulpal exposure, making SRCT the best option for treating deep carious lesions in 18-month follow-ups [8, 9]. However, there is still significant disagreement among dentists regarding the use of SRCT in deep carious lesions extending to the inner third of dentin without obvious potential pulpal exposure. Only 53.7% of the dentists interviewed opted for SRCT in asymptomatic teeth and 44.1% in symptomatic teeth. More continuing education activities and dissemination, with publications of successful clinical cases, can further encourage the use of selective carious tissue removal [10].

The efficacy of selective removal is evidenced by clinical, biochemical, radiographic, microbiological, and histological studies [11]. This technique is cost-effective, patient-friendly, and less sensitive; reduces the risk of pulp exposure; and avoids multiple visits by the patient to the clinic [12]. Thus, this article is aimed at reporting a series of successful cases of SRCT in permanent molars, with follow-up of up to 21 years and the absence of clinical and radiographic signs of the evolution of carious lesions or pulp involvement.

## 2. Case Report

In 2002, a 6-year-old male patient with no previous history of associated systemic diseases was treated in a social partnership project between the city of São Luiz de Paraitinga, São Paulo, Brazil, and the Pierre Fauchard Organization.

**Figure 1:**
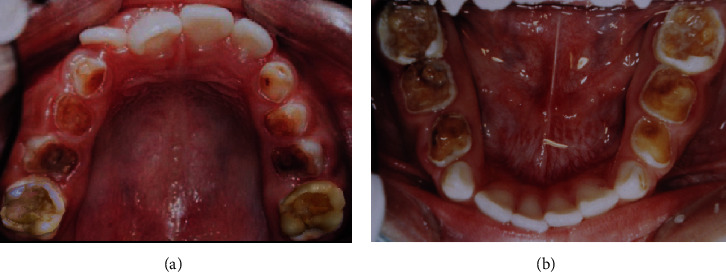
Initial intraoral view in 2002. (a) Upper arch and (b) lower arch.

**Figure 2:**
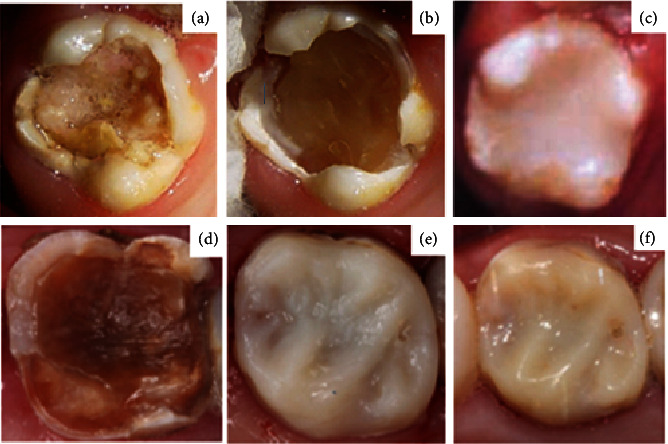
Tooth 16 in 2002: (a) before SRCT treatment, (b) after SRCT treatment, and (c) after GIC restoration. Tooth 16 in 2016: (d) with inactive carious lesion, (e) after composite resin restoration, and (f) at the 21-year follow-up examination.

An initial clinical examination in 2002 revealed carious lesions in the primary and permanent teeth. Teeth 16, 26, 36, and 46 exhibited extensive carious lesions (involving more than three tooth faces), an International Caries Detection and Assessment System (ICDAS) score of 6, absence of spontaneous pain, and sensitivity to percussion (Figure 1). The patient received guidance on oral hygiene and diet. SRCT procedures were performed using manual instruments to prevent pulp exposure, employing the atraumatic restorative treatment (ART) technique. Only the softened dentin was removed. Following SRCT treatment, a thin layer of calcium hydroxide cement (CHC) (Dycal, Dentsply, Pirassununga, SP, Brazil) was applied, and the affected teeth were restored with glass ionomer cement (GIC) (Ketac Molar, 3M ESPE, St. Paul, MN, USA) (Figures 2(a), 2(b), and 2(c) and Table 1). Treatments were conducted under local anesthesia and relative isolation. Only tooth 16 did not require anesthesia.

**Figure 3:**
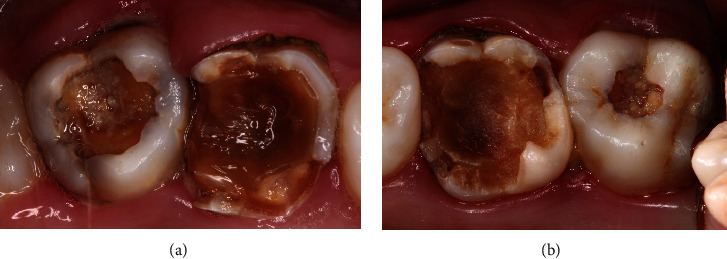
Intraoral view from 2016 with active carious lesions in (a) tooth 17 and (b) tooth 27 and inactive carious lesions in (a) tooth 16 and (b) tooth 26.

**Figure 4:**
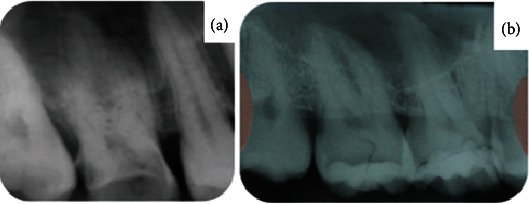
Periapical radiographs of tooth 16 in (a) 2016 and (b) 2023.

**Figure 5:**
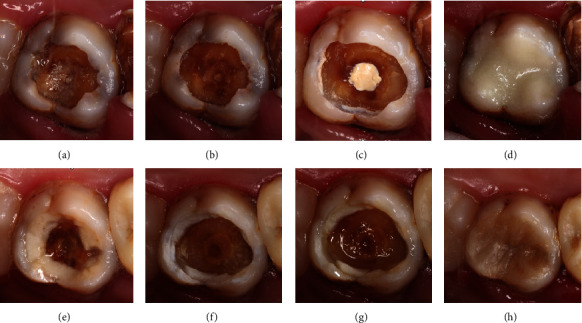
Tooth 17 in 2016 (a) before SRCT treatment, (b) after SRCT treatment, (c) with CHC as a liner material, (d) after restoration with GIC, (e) with partial loss of GIC after 1 year, (f) after removal of residual GIC and preparation of the cavity, (g) after application of the adhesive system, and (h) after restoration with composite resin.

**Figure 6:**
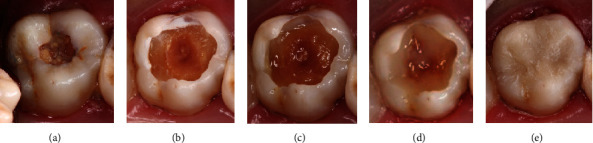
Tooth 27 in 2016 (a) before SRCT treatment, (b) before restoration with composite resin in 2017, (c) after application of the adhesive system, (d) during restorative treatment using the incremental technique, and (e) after restoration.

In a follow-up visit 14 years later, extensive carious lesions were observed in the maxillary second permanent molars, and loss of GIC restorations was noted in the maxillary first permanent molars. There were no reports of spontaneous pain or sensitivity to percussion. The carious lesions of teeth 16 and 26 were inactive. No traces of the previously used restorative or liner materials were noted (Figure 3), and there were no radiographic changes (Figure 4). SRCT treatment was performed on teeth 17 and 27 with manual instruments using the ART technique, and only softened dentin was removed. The teeth were then restored with GIC using CHC as a liner material (Figures 5(a), 5(b), 5(c), and 5(d) and 6).

**Figure 7:**
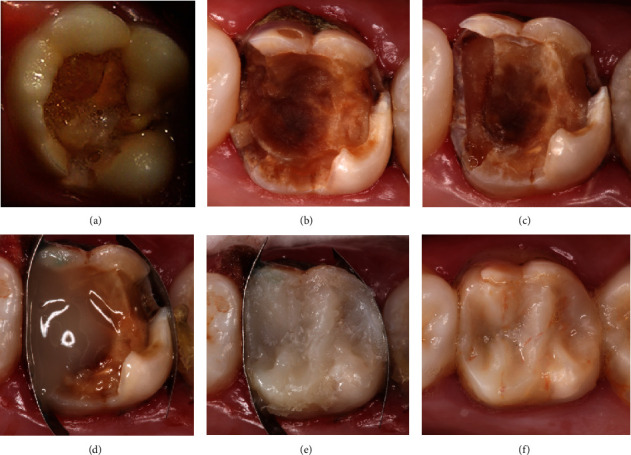
Tooth 26 (a) in 2002 before SRCT treatment, (b) in 2016 with inactive caries lesion, (c) after cavity preparation, (d) during restoration with composite resin, (e) after restoration, and (f) at 21-year follow-up examination.

Teeth 16 and 26 were treated with composite resin (Z350, 3M ESPE, St. Paul, MN, USA) to restore esthetics and function, using the total acid etch technique and a conventional two-step adhesive system (Adper Single Bond 2, 3M ESPE, St. Paul, MN, USA) (Figures 2(d) and 2(e) and 7 and Table 1).

One year later in 2017, the patient returned with a loss of both GIC restorations of the second permanent molars. The teeth were again restored with a composite resin (Opallis, FGM, Joinville, SC, Brazil). An adhesive technique with total acid etching and a conventional two-step adhesive system was used (Ambar, FGM, Joinville, SC, Brazil) (Figures 5(e), 5(f), 5(g), and 5(h) and 6 and Table 1).

**Figure 8:**
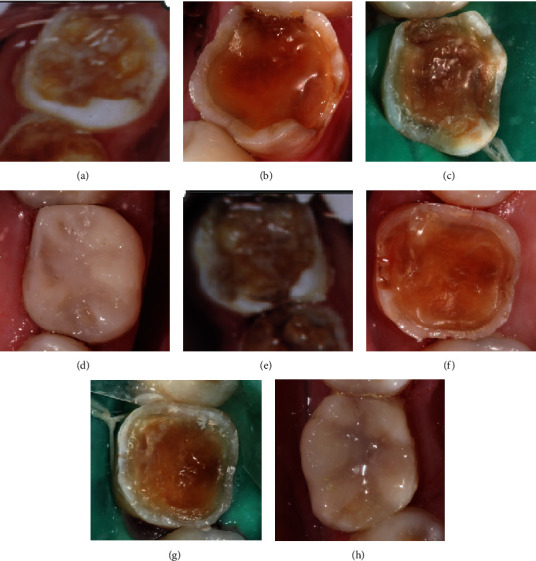
Tooth 36 (a–d) and tooth 46 (e–h) in 2002 (a, e), in 2004 (b, f), in 2021 with inactive lesions (c, g), and after resin restoration (d, h).

In 2021, 19 years after initial treatment, the patient returned with total loss of the GIC and CHC restorations of teeth 36 and 46. The lesions were inactive and had a vitreous appearance (Figures 8(b) and 8(f)). Restorative treatment with composite resin was performed with the total conditioning technique, using a conventional two-step adhesive system under absolute isolation from the operative field (Figure 8 and Table 1).

**Figure 9:**
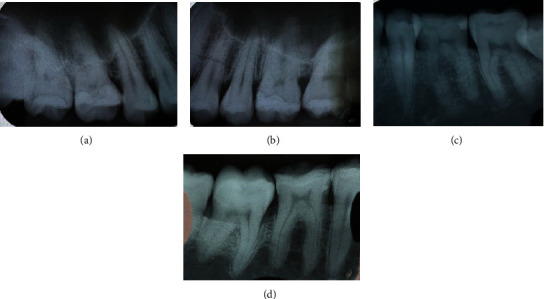
Periapical radiographs of (a) teeth 16 and 17, (b) teeth 26 and 27, (c) tooth 36, and (d) tooth 46 at 21-year SRCT follow-up examination.

**Figure 10:**
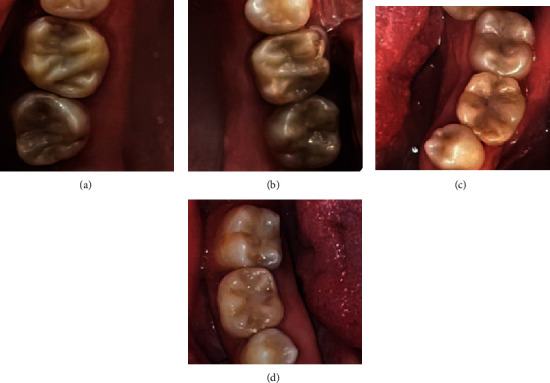
Intraoral view from 2023 with 21-year SRCT follow-up of (a) tooth 16, (b) tooth 26, (c) tooth 36, and (d) tooth 46, as well as 7-year SRCT follow-up of (a) tooth 17 and (b) tooth 27.

The success of the SRCT treatment was determined by the nonprogression of the caries lesions and by the absence of clinical and radiographic signs of pulp alterations after an extended follow-up period (Figures 9 and 10).

## 3. Discussion

The main objective of caries removal is to maintain the tooth and pulp health for as long as possible, following the principles of preserving healthy dental tissue, maintaining pulp vitality, and avoiding exposure and maintaining healthy margins to promote correct sealing of the restoration [13]. Conservative procedures are considered successful when pulp vitality is maintained, and no adverse symptoms are reported after treatment [2]. None of the six reported cases showed clinical signs of pulp alterations after selective caries removal during a 7- to 21-year follow-up.

Strategies for treating deep carious lesions have changed over the years. Complete or nonselective removal of carious tissue is considered overtreatment and is no longer advocated in the literature [8, 9, 13–16]. This change in strategies has resulted from a better understanding of the defensive and restorative response of the pulp-dentin complex to irritation [4].

Khokhar and Tewari [12] recommended CRCT on the surrounding walls, with limited pulp floor and axial wall removal. SRCT in deep cavities should restrict the removal of softened dentin, avoiding exposure or irritation of the pulp. In less deep lesions, removal should reach the firm dentin [13, 17]. The reported cases were treated with SRCT following the international consensus.

Carious lesions are considered profound when they reach the third or the inner fourth of the dentin, as determined radiographically, at the risk of pulp exposure. The maintenance of pulp vitality of these teeth can be achieved with SRCT strategies [4]. Several studies have demonstrated the arresting of the lesion after sealing dentin carious lesions, regardless of the lining material used [2, 7]. Dentin reorganization and its mineral changes are not dependent on the material placed in contact with the carious tissue, suggesting that the host response causes the caries arrest, rather than a material-induced process [7, 18]. CHC was used as a protective liner in a reported case, followed by restoration with high-viscosity GIC. However, according to Alves et al. [19], the CHC liner is unnecessary and does not influence the clinical success of treatment.

Sealing of the carious lesion allows the teeth to biologically respond, isolating the lesion of bacteria from the oral environment and active biofilm [20, 21], arresting the carious process, and allowing time for the defense responses of the dentin-pulp complex. In response to the inflammatory process induced by dental caries, odontoblasts produce a reactionary tertiary dentin matrix. Hardening of the dentin after sealing is a result of tissue remodeling [22]. Arresting dentin caries and hardening of the surface were observed in a reported case.

The study by Ricucci et al. [23] contraindicated SRCT because no histopathological studies support such an indication. However, only eight of the 268 teeth treated and evaluated histologically and bacteriologically were treated with this technique. The presence of bacteria in the tertiary dentin of only two teeth and inflammatory changes in the eight teeth analyzed were observed. However, besides having been evaluated in a small number of teeth, the study did not inform how long after performing the procedure the teeth were extracted and analyzed. The precise evaluation of the preoperative pulp status is an extremely important factor in the success of vital pulp therapy and should also be considered with caution, according to this series of cases in which there was no spontaneous pain and sensitivity to percussion.

Ricucci et al. [23] reported that the bacteria present in the decayed tissue are anaerobic. Leaving decayed dentin near the pulp allows the inflammatory process to lead to pulp necrosis. Limiting bacteria access to dietary sugar can cause them to scavenge protein-shaped nutrients and glycoproteins from demineralized dentin collagen. However, Chibinski et al. [21] observed an increase in metalloproteinases, bone sialoprotein, and collagen 60 days after sealing the cavities, suggesting that these enzymes were secreted to lead to remodeling of the dentin matrix, and not due to caries progression.

According to Ricucci et al. [22], arresting the carious process does not necessarily mean that the bacterial infection is controlled or absent. However, the twelve teeth evaluated histologically and bacteriologically showed no postoperative symptoms or positive response to pulp vitality tests before extraction. Several authors have reported a reduction in bacteria after SRCT and sealing of carious lesion [6, 7, 11, 24, 25]. According to Paddick et al. [26], isolation of the oral carious lesion affects the survival of the microbiota, making it less complex according to phenotypic and genotypic analyses and its composition.

Khokhar and Tewari [12] showed a statistically significant difference in pulp exposure between partial and complete removal of the carious tissue, with a low proportion of exposure in SRCT. The high rate of clinical and radiographic success in SRCT after 18 months suggests that SRCT may be as effective as CRCT in permanent teeth, with the additional advantage of reducing pulp exposure. Incomplete removal has advantages over complete removal, especially in deep lesions [27]. Bjørndal et al. [28] compared nonselective caries removal with S.E. After 5 years, the authors concluded that the S.E. group had a higher number of vital pulps without radiolucency on radiographs.

Verdugo-Paiva et al. [29] evaluated the efficacy and safety of SRCT compared to total removal. With insufficient evidence, they concluded that SRCT may decrease the risk of pulp exposure and the need for endodontics in teeth with deep carious lesions. However, the certainty of the evidence was very low to reduce the risk of the appearance of signs and symptoms of pulp pathology and to achieve a reduction of the risk of failure with the SRCT technique.

In addition, Maltz et al. [30] compared SRCT with S.E. in deep lesions, concerning the maintenance of pulp vitality, for five years. They observed a small amount of necrosis in the SRCT, supporting treatment in a single session. The success rate was 80% for SRCT compared to 56% for S.E. In previous follow-ups of 1.5 years and 3 years, success rates of the SRCT in each case were 99% and 91%, respectively [6, 31]. Treatment in two sessions also has several disadvantages, such as an increase in the cost of treatment, the risk of abandonment of treatment by the patient, failure in the provisional restoration, and the risk of pulp exposure during reintervention. According to Labib et al. [32], success and survival did not differ significantly between SRCT and S.E. after 1 year of this randomized study. The total SRCT costs were similar to those of S.E. performed in two visits. The authors concluded that there is no substantial justification for preferring S.E. over SRCT for lesions extending beyond two-thirds of the dentin, supporting the cases performed in a single session in this series.

Robust evidence supports the use of SRCT for the treatment of carious lesions, especially for deep lesions [16]. However, Schwendicke and Göstemeyer [33] observed that about half of the dentists in most countries in the world did not adopt SRCT for caries treatment. This proportion seems to have decreased in most recent studies. Several factors were found to be associated with the behavior of dentists regarding the removal of carious tissue, such as their age, understanding of caries disease, and its pathogenesis. In the study by Oen et al. [34], only approximately 20% of dentists were in favor of SRCT, indicating that studies on deep caries treatment are necessary. Chevalier et al. [35] reported that students preferred SRCT techniques because they reduced the probability of pulp exposure. Systematically developed behavioral change interventions can be effective in improving the absorption of SRCT [36].

Additionally, future research is needed in order to combine this technique with other features such as low-noise instruments [37] and computerized anesthesia devices [38] in order to understand their mutual effect on caries management.

Thus, from a biological point of view, when treating the tooth as an organ, preserving the portion of the dentin-pulp complex capable of remineralization is essential to maintain its healing capacity [12]. Our study showed the follow-up of six cases of SRCT for up to 21 years. We did not find other reports in the literature with the same follow-up period.

However, one limitation of our study was the small sample size, comprising only six teeth. This limited number of cases may reduce the generalizability of the findings and necessitates caution in interpreting the results. We suggest clinical trials with a longer follow-up period to reaffirm our findings that minimally invasive approaches, particularly those involving selective removal of carious (demineralized) tissue, have been successful.

## 4. Conclusion

No clinical or radiographic alterations were observed after 7 and 21 years of follow-up in permanent molars treated with selective carious tissue removal, supporting the use of this technique.

## Figures and Tables

**Table 1:** Description of the restorative techniques used.

Teeth	Lesion removal	Initial treatment	Final treatment	Follow-up of SRCT
16, 26, 36, 46	SRCT	ART protection with CHC + restoration with GIC	Total acid etching + 2-step adhesive system + composite resin	21 years
17, 27				7 years

## Data Availability

The data that support the findings of this study are available from the corresponding author upon request.
